# Comparison of bone mineral density quantification in dogs using spectral detector computed tomography versus phantom-based conventional computed tomography

**DOI:** 10.3389/fvets.2025.1572887

**Published:** 2025-06-13

**Authors:** Alina Hörmann, Tarek Neubert, Graeme Campbell, Adriano Wang-Leandro, Michael Pees, Christina Strube, Kristina Merhof

**Affiliations:** ^1^Department of Small Animal Medicine and Surgery, University of Veterinary Medicine Hannover, Foundation, Hanover, Germany; ^2^Faculty of Forest Sciences and Forest Ecology, Georg-August University of Göttingen, Göttingen, Germany; ^3^Clinical Science, Philips, Hamburg, Germany; ^4^Department of Small Mammal, Reptile and Avian Medicine and Surgery, University of Veterinary Medicine Hannover, Foundation, Hanover, Germany; ^5^Institute for Parasitology, Centre of Infection Medicine, University of Veterinary Medicine Hannover, Hanover, Germany

**Keywords:** bone mineral density, spectral computed tomography, SDCT, quantitative computed tomography, material decomposition

## Abstract

**Introduction:**

The generation of virtual monoenergetic images using spectral detector computed tomography (SDCT) may facilitate the measurement of bone mineral density (BMD) without the requirement of a phantom. This study has two primary objectives: (1) To compare the BMD values obtained from SDCT maps with those derived from phantom-calibrated values using quantitative computed tomography (QCT) in specific phantom densities and predetermined locations in canine subjects; and (2) to assess the reproducibility, measurement precision, and the potential bias associated with phantom-based measurements.

**Materials and methods:**

SDCT examinations of 49 dogs included a phantom containing four hydroxyapatite inserts. BMD values were manually measured in 18 anatomical locations. A linear model was used to convert Hounsfield units to BMD values (mg/cm^3^). A paired Wilcoxon signed-rank test with Bonferroni-correction and Pearson correlation were used for statistical analysis. A *p*-value of ≤ 0.05 was considered significant.

**Results:**

The statistical analysis demonstrated consistently lower BMD values derived from SDCT data within the phantom volume of interest. However, when compared to QCT, higher BMD values were noted across all anatomical sites. QCT data provided BMD values closer to the density of the phantom, while SDCT data appeared to be less sensitive to phantom positioning and body weight. The absolute differences in phantom values were influenced by the number of voxels without completely correcting the generally observed differences in the measured values.

**Conclusion:**

BMD values from both methods demonstrated significant systematic differences, highlighting the need for further research to optimize SDCT for clinical use.

## 1 Introduction

Bone mineral density (BMD) refers to the mineral concentration in bone (1). Many physiological factors including age, exercise and nutrition have a significant influence on BMD (2–9). Reduced physical activity, such as lameness or immobilization, can decrease BMD and lead to conditions like postoperative implant-induced osteoporosis, especially in toy breed dogs, increasing the risk of refractures after implant removal (10–12). Endocrine disorders, such as hyperparathyroidism and hyperadrenocorticism (13–16), as well as excessive use of steroid anti-inflammatory drugs may cause osteoporosis (13, 17–19). Nutritional deficiencies, particularly in calcium or vitamin D, or those excessively high in phosphorus, can result in metabolic bone diseases (8).

Conventional radiographs are often used in veterinary medicine to detect decreased bone opacity, but at least 30 to 50 percent of the bone mineral content must be diminished to be radiographically apparent (20). Dual-energy X-ray absorptiometry (DEXA) is the gold standard to evaluate BMD in human medicine (21) and offers advantages in cost efficiency, non-invasiveness and low radiation exposure (22). The main disadvantage of DEXA is that it is a two-dimensional (2D) projection technique that measures areal density in g/cm^2^, which can be affected by surrounding overlapping soft tissue (13, 21, 22). Quantitative computed tomography (QCT), a three-dimensional (3D) imaging procedure, is more common in veterinary medicine, allowing the differentiation of cortical and trabecular bone (22, 23). Trabecular bone has been shown to be the metabolically more active tissue compared to cortical bone and is, therefore, more sensitive to changes in bone mass (13, 22).

QCT relates the amount of radiation absorbed by a tissue to a greyscale number called the Hounsfield unit (HU), which reflects the tissue’s physical density. HUs are not constant but strongly energy dependent (24). The HU is a quantitative measurement of radiodensity (25) but lacks direct information on hydroxyapatite (HA) content, the major inorganic component of bone. HA is widely used as a bone-like material for the calibration of DEXA and QCT. True volumetric density measurement in QCT requires external calibration with a HA phantom, often requiring simultaneous scanning of the phantom, which needs prospective study planning since phantoms are not typically included in standard QCT hardware (21, 22). Phantomless techniques are available, based on a synchronous intrinsic calibration using reference measurements in the paraspinal muscle and subcutaneous fat (21, 22).

Based on a simple principle that makes use of the energy-dependent information present in CT images, dual-energy computed tomography (DECT) has been proposed as a tool with greater diagnostic accuracy than DEXA and conventional QCT (22, 26). The same principle but with a different technical approach is used in spectral detector computed tomography (SDCT), which allows data acquisition at multiple photon energy spectra. Using a dual-layer detector, SDCT can distinguish between high-and low-energy photons. The top layer of an yttrium-based garnet scintillator selectively absorbs low-energy photons while the high-energy photons penetrate this layer to reach the bottom layer of gadolinium-oxysulphide (21, 27–31) with the patient being exposed to a conventional poly-spectral x-ray beam only once. SDCT scanners use multiple photon spectra (high and low energy) to separately evaluate the photoelectric effect, which is dominant for photon energies below 100 keV, and the Compton effect, which becomes more important for photon energies above 100 keV. These separate effects are then used to solve for attenuation coefficients, allowing material decomposition and the generation of a variety of spectral images (27, 30–32).

Some materials with similar attenuation in conventional CT can be distinguished by their attenuation at different photon energies in SDCT (21, 29, 30). The application of dual-layer bone densitometry in human medicine and phantom studies has already been demonstrated in a few studies (33–35). SDCT could potentially overcome the disadvantage of QCT by providing a phantomless technique with possible retrospective use of spectral data without prior selection of different scan protocol settings (21, 24).

The objectives of this study were (1) to compare the BMD values from SDCT maps with the reference of HA phantom-calibrated values from QCT in the phantom densities and defined locations in canine patients; and (2) to investigate the reproducibility, internal consistency and bias of the phantom measurement to assess whether spectral data can be used to determine BMD in canine patients without phantom calibration.

We hypothesized that there would be a high level of agreement between volumetric BMD measurements obtained from SDCT and calibrated BMD from HU quantification in QCT, with high internal consistency of BMD measurements from the SDCT data.

## 2 Materials and methods

### 2.1 Study population

In this method-comparative study, a total of 64 dogs were examined for selected criteria between May 2022 and October 2022 at the *Department of Small Animal Medicine of the University of Veterinary Medicine Hannover Foundation*. Third-generation spectral detector computed tomography (SDCT; Philips IQon Spectral CT, Philips Health Care Germany) was utilized, with a HA-phantom (KP 70, Lot Kp 03/3; Scanco Medical AG, Switzerland) manually positioned in the FOV of an entire body or thorax and abdomen scan. The default phantom position was either behind the patient or between the hindlimbs at the level of the tarsal joints, depending on the length of the scan relative to the animal. 34 CT scans of client-owned dogs were conducted for standard diagnostic and monitoring purposes, while images of 30 healthy beagle dogs were used from a former scientific study (36).

Recorded patient data included breed, age, sex, weight, and the reason for presentation.

Exclusion criteria were patients with lameness, known or suspected fractures or increased joint effusion, those with suspected or known aggressive bone lesions and scans with inadequate SDCT image quality. Patients were excluded if the phantom was not fully displayed in all acquisitions, if the FOV did not include the humeral or femoral diaphysis, or if technical errors in reconstruction or data transfer were present.

### 2.2 Density phantom

**Figure 1:**
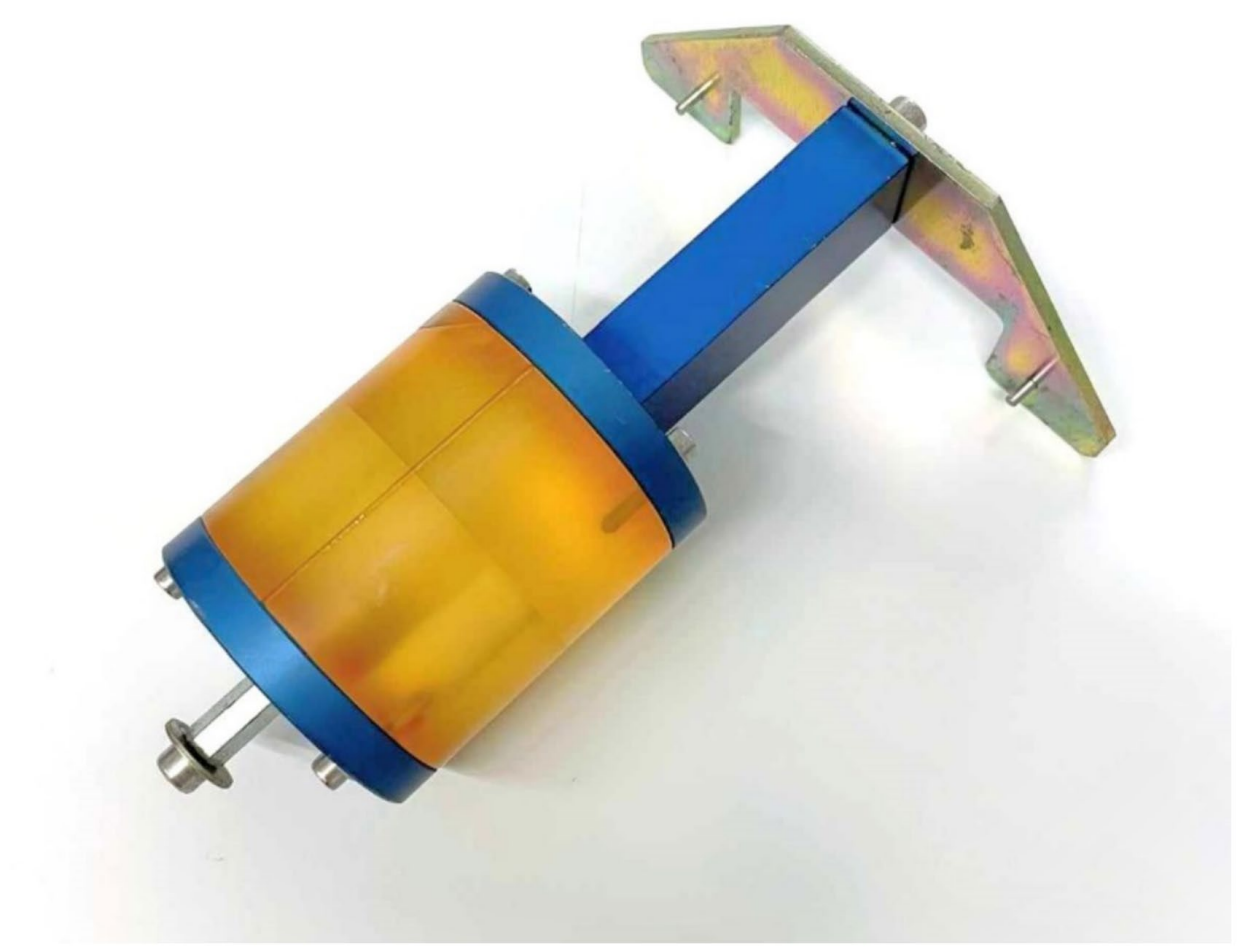
The calcium hydroxyapatite (HA) phantom used in the study, provided by Scanco Medical AG, contains four inserts of known HA density (100, 200, 400, 800 mg HA/cm^3^).

A phantom (KP 70, Lot Kp 03/3) supplied by Scanco Medical AG was included in the patient’s FOV using a third-generation clinical IQon SDCT system (Philips IQon Spectral CT, Philips Health Care Germany) (Figure 1). This cylindrical phantom, with a body length and diameter of 6.5 cm, served as the reference standard. One side contained four cylindrical inserts with uniform HA concentrations in a water-equivalent epoxy resin, with concentrations of 800 mg/cm^3^, 400 mg/cm^3^, 200 mg/cm^3^ and 100 mg/cm^3^ (37).

### 2.3 SDCT image acquisition

The CT scans were conducted for diagnostic purposes in a clinical setting using a SDCT system (SDCT; Philips IQon Spectral CT, Philips Health Care Germany). All patients were scanned in head-first sternal recumbency under general anaesthesia.

SDCT scan protocols were patient-tailored according to the size of the dog and the clinical question being addressed but mostly included a maximum tube potential of 120 kVp and automatic mAs. The helical pitch ranged from 0.5 to 1.5, with a gantry rotation time of 0.5 s. The matrix size was set at 512×512, and slice thickness varied between 0.1–0.3 cm.

Only pre-contrast series were used for the determination of BMD; Conventional 120 kVp images and Spectral Based Images (SBI), including virtual monoenergetic images (40–200 keV images), were reconstructed from raw data. Soft tissue window reconstruction algorithms and BMD maps were used for image analysis.

### 2.4 SDCT image analysis and BMD quantification

Diagnostic discordance was assessed between the two modalities for measuring BMD. Since automatically generated BMD maps are not currently commercially available, two different methods for assessing BMD were conducted:

Analysis of the HU values from conventional reconstructions and calculation of BMD in mg HA/cm^3^ based on spectral material decomposition of monoenergetic (monoE) images. For the latter, virtual monoE images at 50 keV and 200 keV were generated from the SBI datasets, which served as the base images for the material decomposition. Virtual monoE images simulate the appearance of images obtained with a monochromatic X-ray source. They are created by determining the photoelectric and Compton scatter components of the total X-ray attenuation from the high-and low-energy attenuation data of the dual-layer detector (38). The HU values for each keV image are determined via a weighted combination of these components. To set up the algorithm, circular 3D isotropic volumes of interest (VOIs) were drawn in each insert (100–800 mg HA/cm^3^) of the phantom in a sample of ten patient scans, and the HUs in the 50 and 200 keV images were determined. The subsequent generation of the BMD map was based on the mean values of these ten measurements in each of the four phantom inserts. The 50 and 200 keV HU values were plotted against each other to generate a regression line onto which all pixels in the image were projected to calculate the bone volume fraction. The bone fraction in each pixel was then converted into density using the known density values of the phantom and the corresponding bone fractions of the VOIs drawn within the phantom. This resulted in a BMD map in which the value of each pixel represents BMD in mg/cm^3^.

Image analysis was performed using an open-source medical health image processing software (ITK-SNAP 3.6.0, Penn Image Computing and Science Laboratory (PICSL) at the University of Pennsylvania, www.itksnap.org). ITK-SNAP is an interactive software tool for manual and semi-automatic segmentation of 3D medical images (39, 40).

The conventional images and monoE reconstructions at 50 and 200 keV (BMD maps) were synchronized in the software to ensure that measurements were done at the exact same point. BMD values were measured directly in the SBI reconstructed BMD maps, while a conversion of the measured HU value to BMD by the known density of HA in the phantom was necessary for conventional images using the phantom. Drawing a VOI produced a circular, isotropic 3D VOI of equal size and location in each method. For each VOI in conventional images and BMD map, the attenuation value in HU and BMD in mg HA/cm^3^, volume (mm^3^) and number of voxels of the VOI were documented.

**Figure 2:**
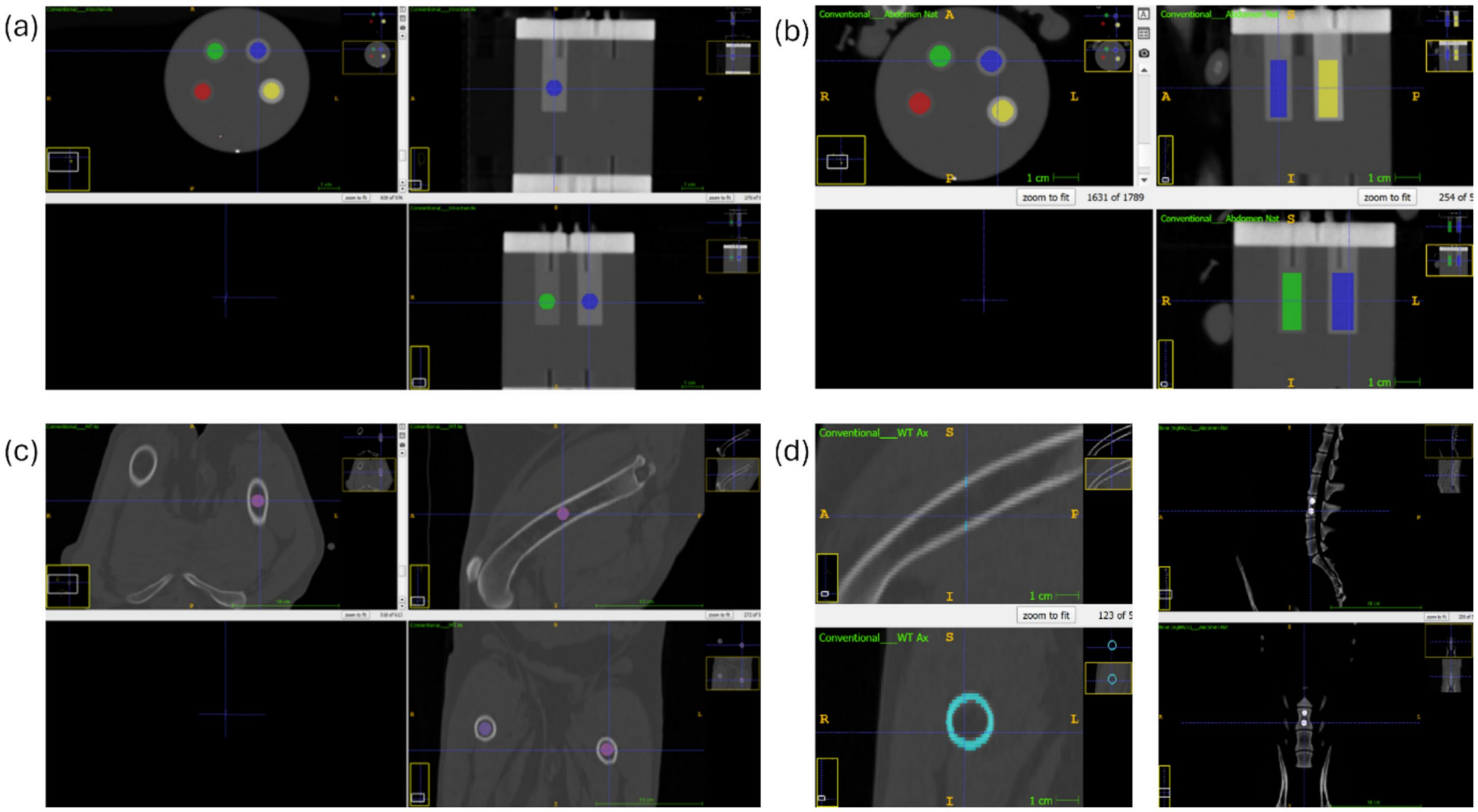
ITK-SNAP-software: **(a)** conventional reconstruction: spherical VOI at the phantom density inserts **(b)** conventional reconstruction: cylindrical VOI at the phantom density inserts **(c)** conventional reconstruction: VOI at the left femoral diaphysis in **(d)** modified image: left side conventional reconstruction: cortical femoral diaphysis; right side SDCT BMD map: VOI at fifth lumbar vertebrae.

A radiologist in training (A.H.), in the third year of a veterinary diagnostic imaging specialisation programme in Germany, manually placed multiple circular VOIs on conventional images using multiplanar reconstructions for orientation. To ensure accurate measurements, the operator was trained at the beginning and assessed the first five patients in consultation with a diagnostic imaging specialist (K.M.). Supervision included the measurements of all further patients and constant availability for clarifications and queries. The largest possible circular VOI was drawn in each location. A VOI was placed centrally in each cylindric insert of the phantom (100–800 mg/cm^3^) (Figure 2a). After the initial data analysis, a second measurement within the phantom inserts was performed to narrow the range of voxel numbers in the phantom VOIs. The scan with the largest voxel size was measured with a cylindrical VOI over the entire length of the phantom inserts. The approximate voxel size of this VOI was used as a guideline for measuring all other phantoms in higher resolution scans. This resulted in cylindrical VOIs of varying lengths within the inserts (Figure 2b). 18 VOIs and one 2D region of interest (ROI) were placed in various bones: the cortical bone of the right femoral mid diaphysis (ROI), the scapula, proximal metaphysis and diaphysis of the humerus, the body of the ilium (cranioventral to the acetabulum, caudal to the gluteal surface), proximal metaphysis and diaphysis of the femur (all measurements bilaterally) and two in the fifth and twelfth thoracic, and fifth lumbar vertebral bodies (T5, T12 and L5). The cortical bone of the femur was delineated in transverse plane with avoidance of the medullary cavity (Figure 2d). All other VOIs were placed in the trabecular compartment, avoiding the cortical bone (Figures 2c,d).

Spherical measurements within the phantom inserts and all localizations with trabecular bone, were performed twice by the same observer for each patient to evaluate reproducibility. The cylindrical phantom measurements and the femoral cortical bone measurements were performed only once as an additional measurement for a more precise evaluation of the data. BMD was assessed using both SDCT BMD maps and conventional data in combination with a HA phantom.

### 2.5 Statistical analysis

All computations were performed using dedicated open-source software (R software, version 4.2.2). Data cleaning, transformation, visualization, and analysis were performed using R Studio (2023.12.0, R Core Team, 2023, R Foundation for Statistical Computing). The Jarque-Bera-Test was used to evaluate the normality of the data, which were found to be non-normally distributed.

The intraclass correlation coefficient (ICC) was calculated for each method to assess the agreement of repeated measurements within individual patients, separately for both the conventional and SBI datasets. Data from each method, the calibrated QCT data and unmodified BMD map values from the SBI data were compared using Tukey’s *post hoc* tests. For comparison of the data, a linear model was applied using the HU values of the known phantom density inserts to convert the HU value of all VOIs to a calcium-HA concentration (mg/cm^3^) for VOIs within the phantom. All HU values were linearly scaled for each individual patient separately. The factors between HU and calcium-HA concentration (mg/cm^3^) were calculated to scale the remaining conventional data with the linear model for each patient. Diagnostic discordance was assessed between the unmodified BMD map values (mg HA/mg^3^) and the converted HU BMD values.

A paired Wilcoxon signed-rank test with Bonferroni-corrected adjusted *p*-values was used to compare the measurement methods for VOIs within the phantom and the patient. Pearson correlation coefficients were calculated to assess the relationship between the number of voxels and body weight, as well as measurement methods and body weight. Significance was set at *p* ≤ 0.05 for all tests.

## 3 Results

### 3.1 Signalment and clinical findings

A total of 49 dogs (*N* = 49) met the inclusion criteria. Patients included Beagle dogs (*n* = 25), mixed-breed dogs (*n* = 6), Labrador Retrievers (*n* = 3), Boxers (*n* = 2), Siberian Huskies (*n* = 2), Entlebucher Mountain dog (*n* = 1), French Bulldog (*n* = 1), German Hound (*n* = 1), German Shepherd (*n* = 1), Golden Retriever (*n* = 1), Jack Russel Terrier (*n* = 1), Parson Russel Terrier (*n* = 1), Rhodesian Ridgeback (*n* = 1), Saluki (*n* = 1), Spaniel (*n* = 1), Yorkshire Terrier (*n* = 1). The age of the patients ranged from 1.26–16.5 years with a mean age of 5.09 ± 4.67 years. The weights ranged from 2.2 to 54.7 kg, with a mean weight of 14.53 ± 11.65 kg. There were 19 intact females, five spayed females, 20 intact males and five castrated males.

### 3.2 BMD assessment

The intraclass correlation coefficient (ICC) was 1.00 for both the conventional and SBI methods, indicating perfect agreement of repeated measurements within individual patients. This confirms that manual placement of the VOIs was consistent and precise across repetitions.

**Figure 3:**
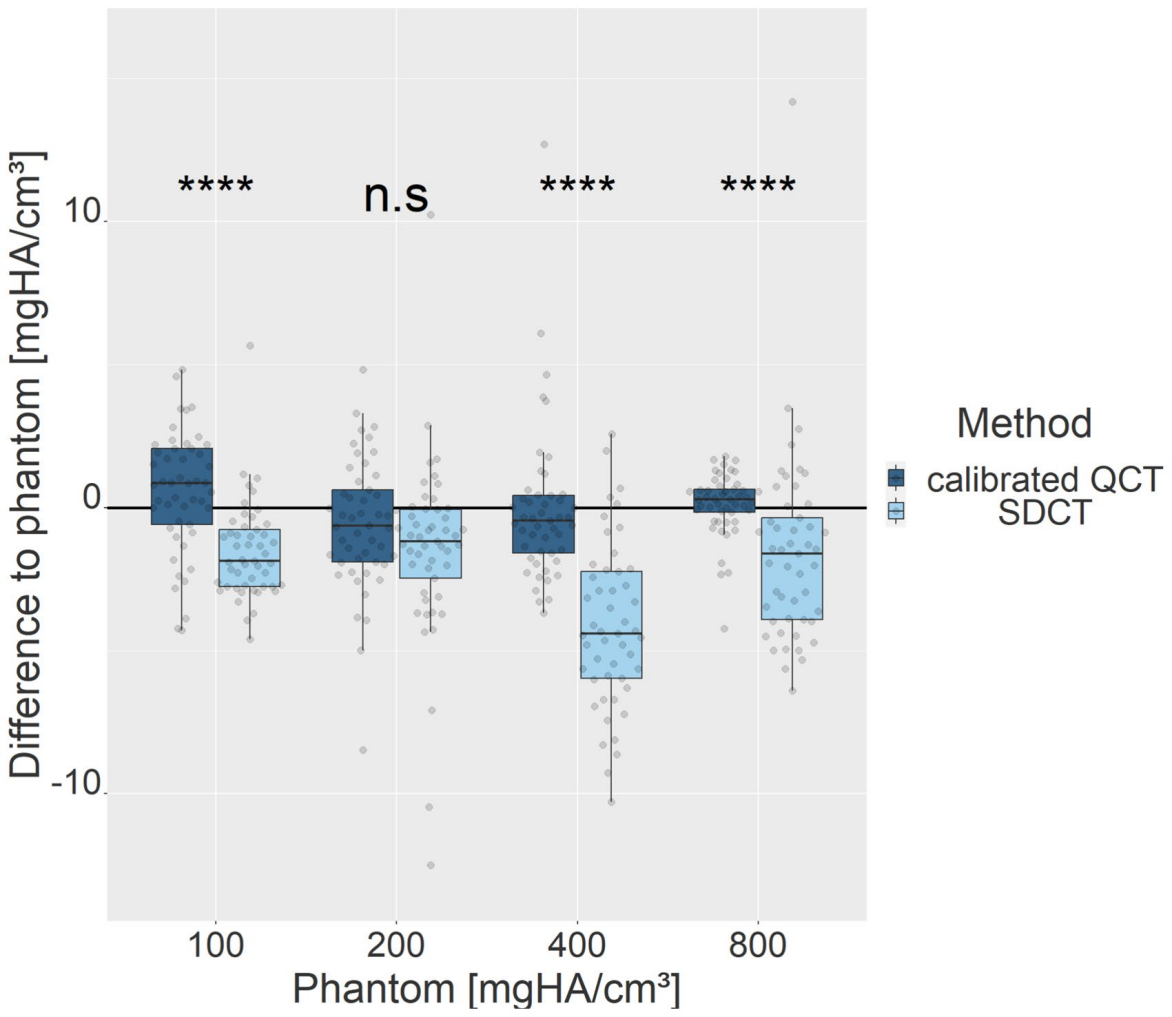
Deviations of measurement methods relative to the phantom values for each density insert (100, 200, 400, 800 mg HA/cm^3^). Light blue represents SDCT values, while dark blue denotes calibrated QCT values. The zero line indicates the phantom reference values. ****denotes a level of significance of *p* ≤ 0.0001, ns = non-significant.

A linear model for the transformation of HU values to calcium-HA concentrations applied for comparability between QCT and SDCT data revealed statistically significant differences for three of the four density inserts (padj = 2.692e-07 for 100 mg/cm^3^, padj = 0.364 for 200 mg/cm^3^, padj = 3.324e-09 for 400 mg/cm^3^, padj = 2.264e-05 for 800 mg/cm^3^; *p*-value after adjustment for the multiple comparisons with Bonferroni correction; Figure 3). Measurement errors were significantly smaller for QCT compared to SDCT across all densities. Calibrated conventional values were closer to the phantom value and, therefore, more accurate in all phantom inserts (mean of differences of calibrated QCT values to the phantom ±SD: 100 mg/cm^3^ = 1.75 (±) 1.30; 200 mg/cm^3^ = 1.79 (±) 1.55; 400 mg/cm^3^ = 1.73 (±) 2.09; 800 mg/cm^3^ = 0.795 (±) 0.782). Measurements of SDCT data were lower for all density inserts (mean of differences of SDCT values to the phantom: 100 mg/cm^3^ = 1.92 (±) 1.21; 200 mg/cm^3^ = 2.29 (±) 2.68; 400 mg/cm^3^ = 4.36 (±) 2.50; 800 mg/cm^3^ = 2.78 (±) 2.37). Notably, the 400 mg/cm^3^ insert in SDCT showed the highest negative deviation.

**Figure 4:**
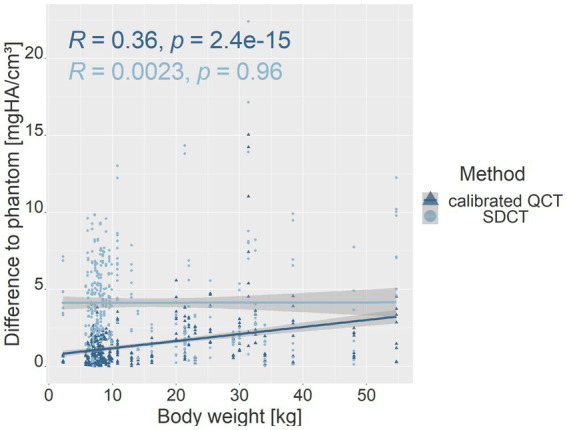
Scatter plot showing the relationship between phantom value deviations and body weight for calibrated QCT (dark blue) and SDCT (light blue) values. The *y*-axis represents the difference in mean values for all dogs, relative to each phantom density (mg HA/cm^3^), while the *x*-axis shows body weight (kg). R represents the Pearson correlation coefficient; p represents the *p*-value (*p*-value ≤ 0.05).

Correlation analysis between phantom values of each measurement method and body weight indicated a positive, moderate correlation between the calibrated QCT data and body weight (*R* = 0.36, *p* = 2.4e-15; *p*-value ≤ 0.05), while no significant correlation was found between the SDCT data and the body weight (*R* = 0.0023, *p* = 0.96; p-value ≤ 0.05; Figure 4). Phantom positioning inconsistencies in the FOV, due to the different sizes of the dogs, were also tested as an influencing factor on the results. When excluding seven dogs with variable phantom positioning, the QCT values remained stable (*R* = 0.35, *p* = 1.7e-11; *p*-value ≤ 0.05), whereas SDCT results showed a slight negative correlation (*R* = −0.21, *p* = 9.7e-05; *p*-value ≤ 0.05), suggesting a small impact of phantom positioning on spectral data accuracy.

**Figure 5:**
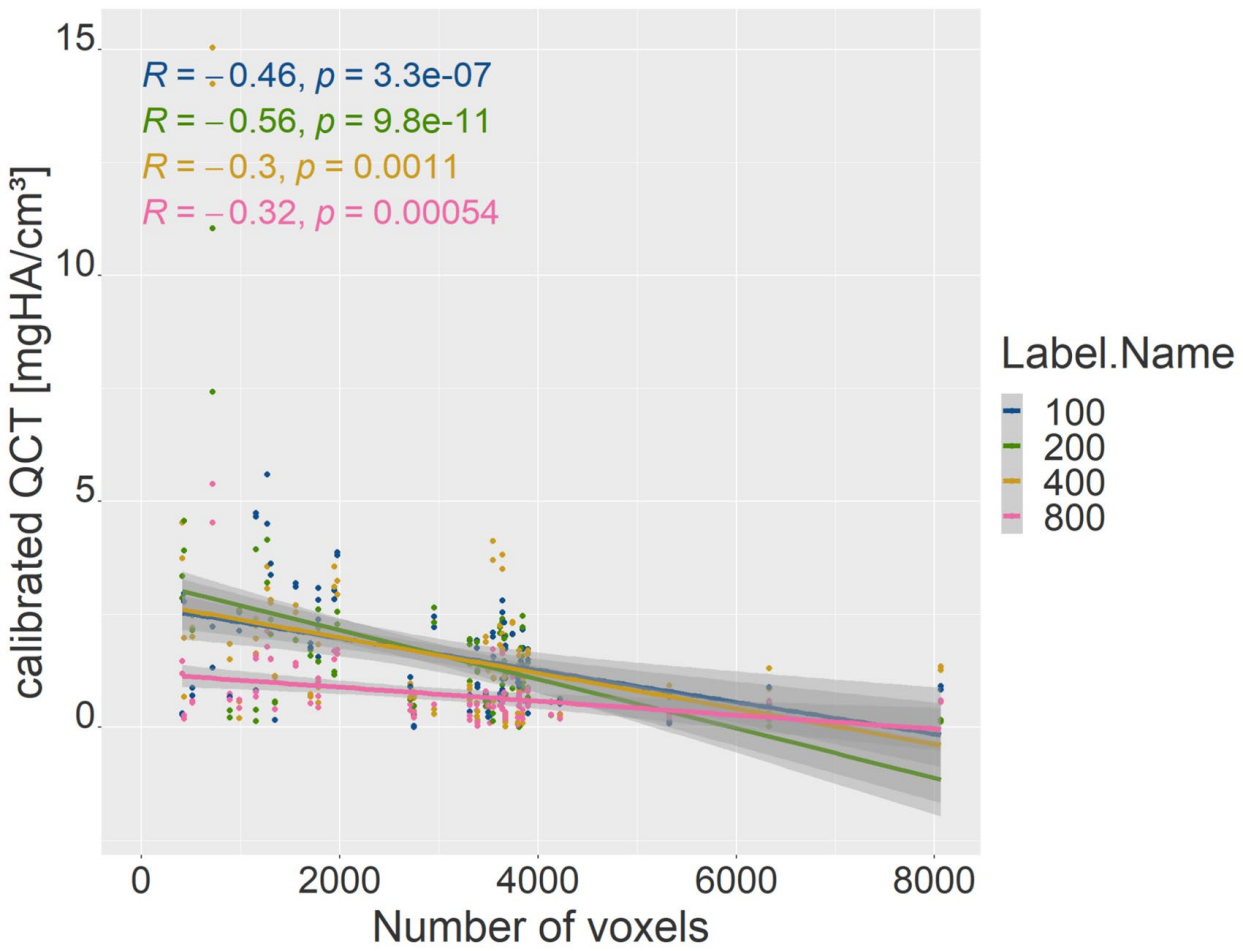
Correlation between voxel count (*x*-axis) and the absolute difference of mean calibrated QCT values for each phantom density (*y*-axis). The blue line represents the 100 mg HA/cm^3^ insert, green the 200 mg HA/cm^3^ insert, yellow the 400 mg HA/cm^3^ insert, and pink the 800 mg HA/cm^3^ insert. *R* represents the Pearson correlation coefficient; *p* represents the *p*-value (*p*-value ≤ 0.05).

**Figure 6:**
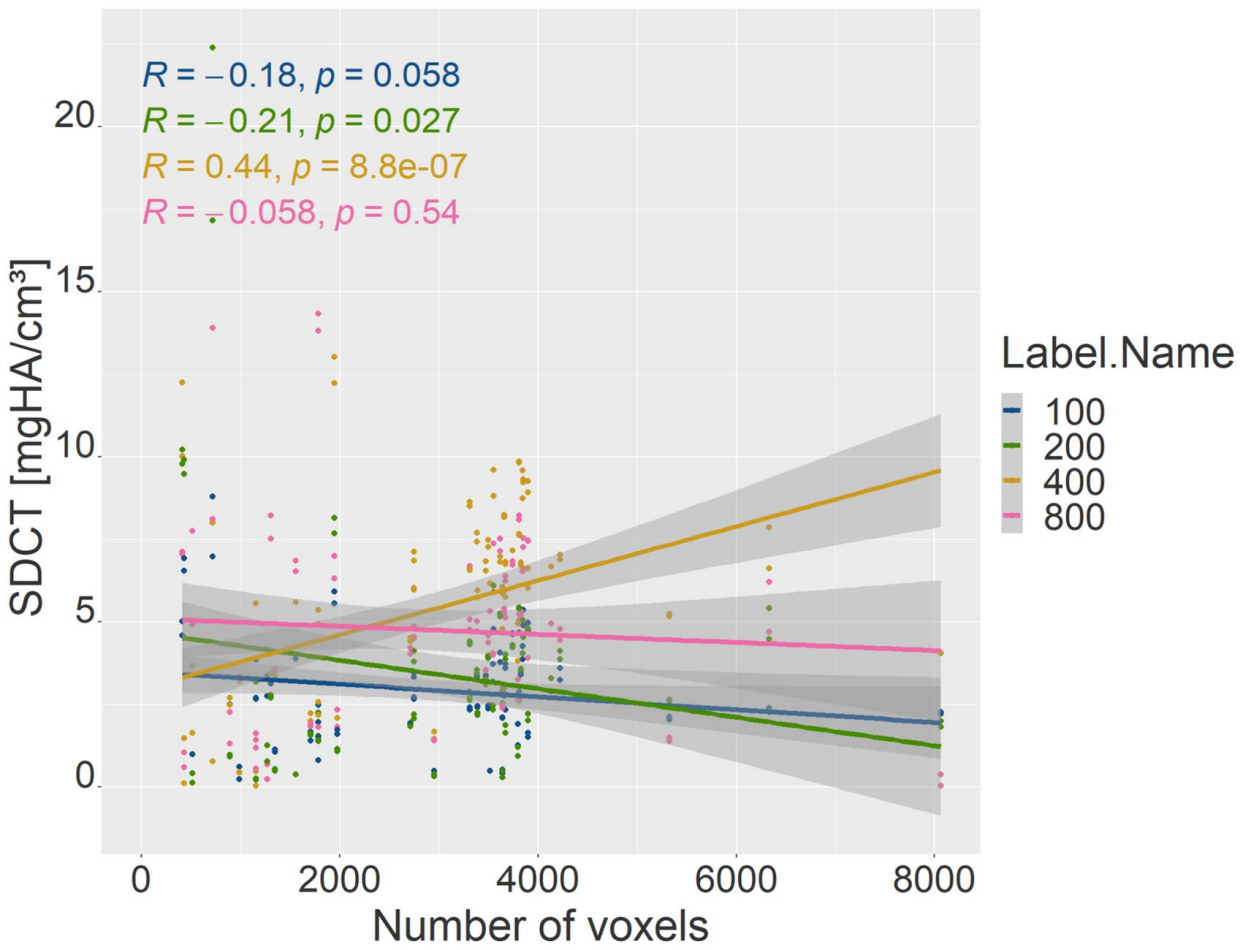
Correlation between voxel count (*x*-axis) and the absolute difference of mean SDCT values for each phantom density (*y*-axis). The blue line represents the 100 mg HA/cm^3^ insert, green the 200 mg HA/cm^3^ insert, yellow the 400 mg HA/cm^3^ insert, and pink the 800 mg HA/cm^3^ insert. R represents the Pearson correlation coefficient; *p* represents the *p*-value (*p*-value ≤ 0.05).

**Figure 7:**
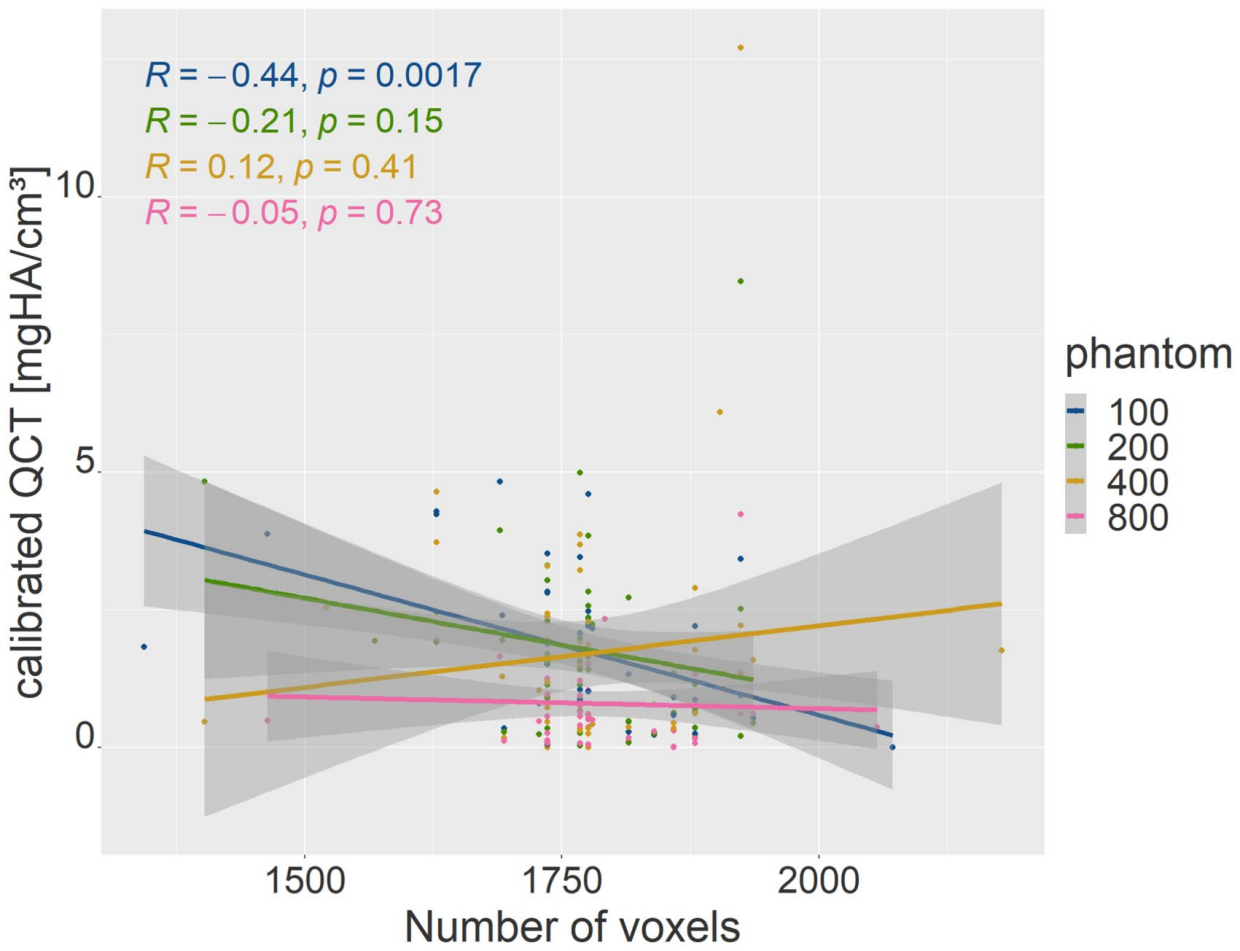
The plot is similar to Figure 5 with lower voxel range after cylindrical measurement. Correlation between voxel count (*x*-axis) and the absolute difference of mean calibrated QCT values for each phantom density (*y*-axis).

**Figure 8:**
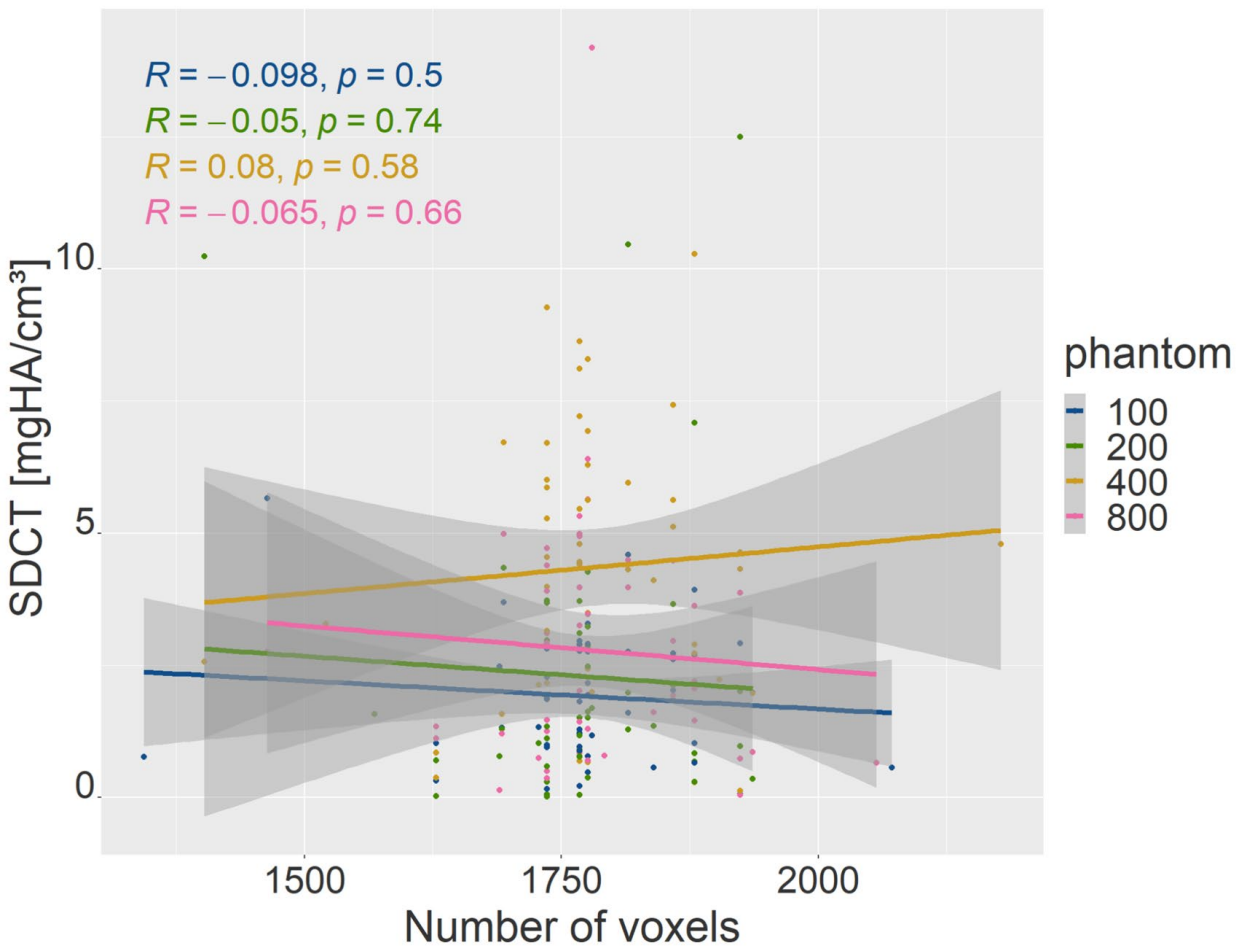
The plot is similar to Figure 6 with lower voxel range after cylindrical measurement. Correlation between voxel count (*x*-axis) and the absolute difference of mean SDCT values for each phantom density (*y*-axis).

The number of voxels used in VOI measurements in the phantom densities ranged from 416 to 8,064. There was a strong negative correlation between the number of voxels and patient body weight (*R* = −0.77, *p* = <2.2e-16; *p*-value ≤ 0.05). Therefore, the absolute deviation of the mean phantom QCT values for each density was evaluated in relation to the number of voxels (Figure 5). The higher the number of voxels in the VOI of the phantom measurement, which was evident in smaller dogs, the less deviation was detected from the mean phantom values (*R* = −0.46, *p* = 3.3e-07 for 100 mg HA/cm^3^, *R* = −0.56, *p* = 9.8e-11 for 200 mg HA/cm^3^, *R* = −0.3, *p* = 0.0011 for 400 mg HA/cm^3^, *R* = −0.32, *p* = 0.00054 for 800 mg HA/cm^3^; *p*-value ≤ 0.05). The 800 mg HA/cm^3^ insert calibrated QCT values were the most consistent. The SDCT data for the 100, 200, and 800 mg HA/cm^3^ inserts, a negative correlation indicated improved accuracy with increasing number of voxels (*R* = −0.18, *p* = 0.058 for 100 mg HA/cm^3^, *R* = −0.21, *p* = 0.027 for 200 mg HA/cm^3^, *R* = −0.058, *p* = 0.54 for 800 mg HA/cm^3^; *p*-value ≤ 0.05; Figure 6). In contrast, accuracy decreased as voxel count increased for the 400 mg HA/cm^3^ insert, showing a positive correlation (*R* = 0.44, *p* = 8.8e-07; *p*-value ≤ 0.05; Figure 6). The cylindrical VOIs within the phantom, which exhibit a more uniform voxel distribution, demonstrated a voxel count range of 1,403 to 2,178 voxels. Significance was found only for the 100 mg/cm^3^ insert in the calibrated QCT data (*R* = −0.44, *p* = 0.0017 for 100 mg HA/cm^3^; Figure 7). All other measured values no longer showed any significance with an adjusted voxel count (Figures 7, 8). When comparing spherical and cylindrical phantom measurements, the highest deviation from the phantom value remains constant in the 400 mg HA/cm^3^ insert of the SDCT data (Figures 6, 8).

**Figure 9:**
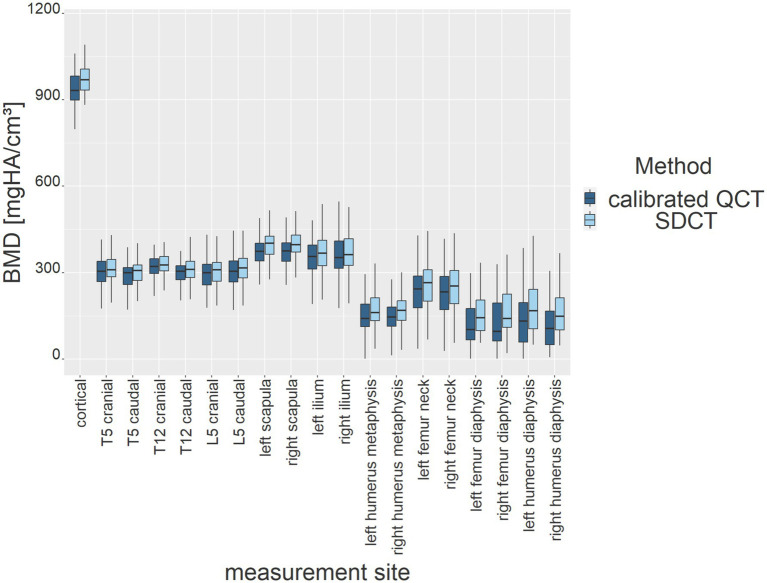
BMD results (*y*-axis) of both measurement methods for all dogs at each anatomical measurement site (*x*-axis). Results are presented as box plots (dark blue for calibrated QCT, light blue for SDCT).

Overall, the measurement methods differed across various anatomical localizations (*p*-value < 2.2e-16; p-value ≤ 0.05). It was found that the SDCT BMD values were consistently higher than those of calibrated QCT (BMD SDCT mean = 263.47 mg/cm^3^; range = 0.0–864.51; BMD calibrated QCT mean = 242.783 mg/cm^3^; range = 0.42–507.09). Figure 9 and Table 1 illustrate that SDCT values consistently showed higher median values and interquartile ranges (IQR) for all dogs and anatomical sites compared to QCT. The results show no significant difference for the localizations in the vertebral bodies, the ilium and the femoral neck. All other measurement sites showed differences of varying degrees of significance, which are listed in Table 1. The largest differences in BMD measurements between the two methods were observed in the humeral and femoral diaphyseal regions.

**Table 1:** Median and interquartile range (IQR) for both measurement methods at each anatomical measurement site.

Measurement site	Median QCT	Median SDCT	IQR QCT	IQR SDCT	Adj. *p*-value	Level of significance
Cortical	932.23	969.86	95.48	93.86	0.34	*
T5 cranial	303.93	310.02	86.22	66.99	1	ns
T5 caudal	298.99	307.44	70.98	66.09	1	ns
T12 cranial	320.80	326.72	66.39	60.10	1	ns
T12 caudal	304.91	310.45	75.28	77.54	1	ns
L5 cranial	299.35	310.07	80.29	78.17	1	ns
L5 caudal	304.69	315.68	69.98	71.23	1	ns
Left scapula	373.14	401.41	86.36	80.59	0.038	**
Right scapula	374.89	396.53	87.94	90.54	0.057	**
Left ilium	355.31	367.12	103.29	103.36	1	ns
Right ilium	352.42	362.15	115.23	109.56	1	ns
Left humerus metaphysis	140.83	161.01	76.35	82.43	0.095	**
Right humerus metaphysis	145.94	168.33	68.35	64.47	0.114	**
Left femur neck	242.89	237.88	125.23	112.55	1	ns
Right femur neck	232.75	253.28	126.30	125.90	1	ns
Left femur diaphysis	102.69	143.15	111.35	107.87	0.057	**
Right femur diaphysis	95.85	140.68	132.34	119.46	0.038	**
Left humerus diaphysis	131.16	167.78	135.23	142.87	0.057	**
Right humerus diaphysis	105.69	148.87	117.90	115.34	0.012	***

Adjusted *p*-value after Bonferroni-correction for absolute differences between measurement methods. Denotation of the level of significance: ns = non-significant, * = 0.05 > *p* > 0.01, ** = 0.01 > *p* > 0.001, *** = *p* < 0.01.

**Figure 10:**
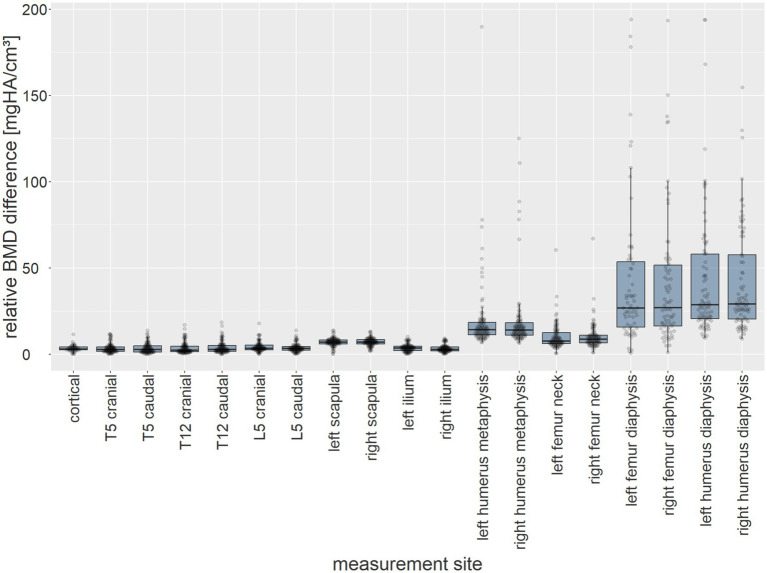
The relative BMD difference between measurement methods (mg HA/cm^3^) of all dogs (*y*-axis) at each anatomical measurement site (*x*-axis) presented as box plots.

The relative BMD difference in bone density measurements was presented, as the absolute values may need to be normalized against the total bone density (Figure 10).

## 4 Discussion

The results of our study provide important insights into the measurement accuracy, reproducibility, and potential biases of BMD maps in SDCT. A comparison with calibrated QCT as a reference standard highlights significant differences between the two methods in both phantom-and patient-based measurements. Results indicated that spectral data values were consistently lower for all phantom inserts and higher in all patient VOIs when compared to calibrated QCT data.

Precise manual drawing of VOIs ensures consistency and reproducibility. BMD quantification methods, supporting the reliability of both quantification methods. Although intra-observer variability was low, indicating consistent results when the same observer repeated measurements, it is important to note that inter-observer variability was not assessed, which may limit the generalisability of the results across different evaluators.

Compared to the calibrated phantom-based QCT, SDCT values are less accurate. Comparing VOIs within the phantom, values of calibrated QCT revealed closer proximity and greater accuracy across all density inserts, indicating that measurement bias is significantly greater with SDCT than with QCT. Marked deviations in the 400 mg/cm^3^ insert show up as strong outliers, suggesting that SDCT may be less reliable for mid-range density measurements than QCT.

Variations in measurement accuracy evident between SDCT and QCT can be attributed to several factors. As the study was conducted in a clinical setting, scanning parameters were tailored depending on the patient and the clinical indication. The position of the phantom in the FOV and the number of voxels were not the only statistical influences on the differences in the data. A limitation of the clinical setting is the non-uniform parameters. A non-uniform table height or the distance of the patient or, in this study, the phantom to the isocentre have already been described as influencing the CT number (41, 42). In general, the accuracy of the values for all phantom densities improved slightly in proportion to the number of voxels in the VOI but still showed the same discrepancies. In computed tomography, the voxel size in a region of interest depends on both the pixel size (x-y plane) and the slice thickness (z-axis). The pixel size can be described by the following equation: Pixel size = FOV/matrix size. Any change in these two parameters changes the CT image resolution or voxel size (43). The discrepancies were influenced by body weight, voxel count and phantom positioning. Heavier dogs were scanned with a larger slice thickness. This, combined with the larger FOV in larger bodies, results in larger and fewer voxels. Higher precision with larger VOI makes VOIs make the VOI a critical factor for BMD measurement, consistent with the results of Tagucchi et al. (44). One limitation that is mitigated in the cylindrical phantom measurement with a more uniform voxel distribution is the unequal number of patients with varying body weights. Consequently, the results from the first measurement, using spherical VOIs, may be influenced by a small subset of patients with either very low or very high body weight.

In this study, BMD values measured by SDCT were consistently lower for all inserts compared to that of the corresponding calibrated QCT data, aligning with prior findings in human medicine (33, 34, 45). However, methodological differences exist between studies. Previous research in human medicine on virtual monoE images suggests that BMD underestimation in spectral CT (e.g., dual-energy or spectral photon-counting CT) compared to QCT can arises from variations in imaging techniques. MonoE images at high and low keV settings were used for material decomposition. High keV settings, while reducing noise, may smooth fine details, causing underestimation of BMD (35, 46). Conversely, low keV settings enhance contrast but amplify noise, potentially leading to overestimation of BMD values (46). The energy-dependent nature of virtual monoE imaging can reduce beam hardening artifacts, although low keV levels may exaggerate calcium contrast, leading to overestimation, while high keV levels may underestimate attenuation, causing BMD underestimation. In contrast, QCT relies on polychromatic X-rays, making it more susceptible to beam hardening artifacts, unless corrected during reconstruction (46). However, the monochromatic image formation in spectral CT, conducted on the raw data, effectively eliminates these artifacts, as noted by van Hamersvelt et al. (47). Additionally, lower spatial resolution in QCT compared to SDCT can lead to partial volume averaging, often yielding higher BMD estimates in trabecular bone. Hofmann et al. further reported that QCT may overestimate HU scores compared to their methods and the American College of Radiology guidelines, suggesting that dual-energy CT provides superior accuracy compared to QCT (48).

The finding of consistently higher BMD values in SDCT compared to calibrated QCT across all anatomical locations contrasts with the negative differences when compared to phantom densities. Higher BMD values obtained with SDCT, relative to QCT *in vivo* are consistent with studies using fat-free phantoms in dual-source CT (33, 45, 49). A tendency for QCT-derived BMD to be underestimated is more likely than an overestimation of the spectral data, as the fat-related error is well-known in clinical practice (33, 50, 51). This is mirrored by the largest deviations, particularly notable in the diaphysis, which have the highest fat content. Kuiper et al. reported measurement errors in QCT bone mineral measurements ranging from 7.2 to 25.3%, due to the variable marrow fat content in the femoral neck of humans (52). A study examining the fat content of the femurs of normal adult animals found that the average fat content of the bone marrow was more than 80% (53).

The argument regarding the fat-related error is undermined by the observation that all locations within the patient demonstrate higher SDCT values, with the smallest differences occurring not at cortical locations, but at the vertebral bodies. It is more plausible that the SDCT values are consistently higher or that the overestimation is more pronounced in specific regions, particularly the diaphyseal area or potentially in regions with higher fat content. This suggests that the observed discrepancies are more likely attributable to a systematic error related to the patient’s anatomy, rather than to the phantom model. One hypothesis is that material decompensation may be more effective in the range of 200–300 mg HA/cm^3^, possibly due to the specific composition of water and bone components, as compared to regions containing higher fat or blood content. Three-material decomposition, along with the quantification of fat in bone marrow and liver tissue using dual-energy techniques, presents additional opportunities to account for this factor, which was not considered in the design of this study (48, 54–56).

Our study had several limitations but provides a foundation for future research to integrate these factors into the study planning and establish standardized protocols for clinical use. Both SDCT and QCT are influenced by partial volume artifacts and patient factors, like breed, patient size, age, body weight, and body mass index, which were not accounted for in this study, and warrant further investigation. Pathologies affecting BMD, such as hyperadrenocorticism, were not excluded, as VOIs with altered bone density should be comparable between both methods. Therefore, the results cannot be used as reference values for canines, for which such standards are lacking.

The advantage of spectral BMD maps compared to a phantom-based method is that data is acquired without preselected examination protocols and can, therefore, be used retrospectively. The method used in the present study requires a negligible amount of training and time. The development of software that enables real-time bone density measurement opens the door to obtaining immediate relevant clinical information in multiple pathologic conditions, such as endocrinopathies and metabolic bone disease.

Despite the widespread use of BMD quantification through QCT *in vivo*, SDCT should be benchmarked against more sensitive methods, such as micro-CT, bone histomorphometry, or ex vivo analysis of burned and chemically analyzed bone samples for better result interpretation. Guha et al. (57) reported that QCT consistently overestimated microstructural parameters compared to micro-CT across multiple anatomical sites. Similarly, Wagner et al. found that although QCT provided an unbiased estimate of ash weight in the femoral neck, it underestimated ash density, indicating limitations in accurately capturing local density and bone microarchitecture (58). However, these reference methods are not applicable to our in vivo patient cohort.

Overall, further studies are needed to integrate this technique, which we believe has the potential to become established as a retrospective, phantomless BMD measurement technique, in a daily clinical setting routine.

In conclusion, BMD values from SDCT and calibrated QCT show significant differences and further studies on larger populations are necessary to address the factors affecting measurement accuracy and develop standardized protocols for clinical use.

## Data Availability

The original contributions presented in the study are included in the article/supplementary material, further inquiries can be directed to the corresponding author.
